# Live imaging of collagen deposition during skin development and repair in a collagen I – GFP fusion transgenic zebrafish line

**DOI:** 10.1016/j.ydbio.2018.06.001

**Published:** 2018-09-01

**Authors:** Josephine L. Morris, Stephen J. Cross, Yinhui Lu, Karl E. Kadler, Yongbo Lu, Sarah L. Dallas, Paul Martin

**Affiliations:** a School of Biochemistry, Faculty of Biomedical Sciences, University Walk, University of Bristol, Bristol, BS8 1TD, UK; b Wolfson Bioimaging Facility, Faculty of Biomedical Sciences, University Walk, University of Bristol, Bristol, BS8 1TD, UK; c Wellcome Trust Centre for Cell-Matrix Research, Faculty of Biology, Medicine and Health, The University of Manchester, Michael Smith Building, Oxford Road, Manchester, M13 9PT, UK; d Department of Oral and Craniofacial Sciences, School of Dentistry, University of Missouri-Kansas City, Kansas City, Missouri, United States; e Department of Biomedical Sciences, Texas A&M University College of Dentistry, 3302 Gaston Ave., Dallas, TX 75246, United States; f School of Physiology, Pharmacology and Neuroscience, Faculty of Biomedical Sciences, University Walk, University of Bristol, Bristol, BS8 1TD, UK; g School of Medicine, Cardiff University, Cardiff, CF14 4XN, UK

**Keywords:** Collagen-I, Zebrafish larvae, Live imaging, Skin, Wound healing

## Abstract

Fibrillar collagen is a major component of many tissues but has been difficult to image in vivo using transgenic approaches because of problems associated with establishing cells and organisms that generate GFP-fusion collagens that can polymerise into functional fibrils. Here we have developed and characterised GFP and mCherry collagen-I fusion zebrafish lines with basal epidermal-specific expression. We use these lines to reveal the dynamic nature of collagen-I fibril deposition beneath the developing embryonic epidermis, as well as the repair of this collagen meshwork following wounding. Transmission electron microscope studies show that these transgenic lines faithfully reproduce the collagen ultrastructure present in wild type larval skin. During skin development we show that collagen I is deposited by basal epidermal cells initially in fine filaments that are largely randomly orientated but are subsequently aligned into a cross-hatch, orthogonal sub-epithelial network by embryonic day 4. Following skin wounding, we see that sub-epidermal collagen is re-established in the denuded domain, initially as randomly orientated wisps that subsequently become bonded to the undamaged collagen and aligned in a way that recapitulates developmental deposition of sub-epidermal collagen. Crossing our GFP-collagen line against one with tdTomato marking basal epidermal cell membranes reveals how much more rapidly wound re-epithelialisation occurs compared to the re-deposition of collagen beneath the healed epidermis. By use of other tissue specific drivers it will be possible to establish zebrafish lines to enable live imaging of collagen deposition and its remodelling in various other organs in health and disease.

## Highlights

• A GFP-collagen I transgenic zebrafish has been generated for live, in vivo, imaging.
• Collagen fibrils are initially deposited randomly beneath the developing epidermis.
• This random collagen array subsequently becomes orthogonally aligned.
• Collagen I deposition following larval wounding recapitulates developmental deposition.
• Expression of GFP-collagen enables study of collagen dynamics in health and disease.

## 1 Introduction

Collagen I is a major extracellular matrix (ECM) component within the sub-epithelial, dermal layer of the skin, where it provides structural support and acts as both a substrate for cell adhesion and migration (Van Goethem et al., 2010), and as an activator of several signalling cascades (Leitinger and Hohenester, 2007). When skin is damaged one of the key steps during repair is the deposition of ECM, in particular collagen I, to form granulation tissue which functions as a temporary replacement for the damaged dermal and sub-dermal tissues. Aberrant collagen deposition in mammalian adult skin repair leads to scarring, which can be debilitating for subsequent tissue function (Eming et al., 2014). By contrast, wounding of zebrafish skin leads to deposition of a collagen “scar” which is then subsequently resolved (Richardson et al., 2013) and this may provide an ideal model to determine how collagen deposition at wound sites might be modulated to prevent fibrosis and scar formation. Here we report a zebrafish line expressing fluorescent collagen that can assemble into fibrils and provides opportunities to visualise the dynamics of collagen deposition in vivo.

Previous electron microscope studies in mouse and chick have revealed some of the cellular mechanisms of collagen I deposition, and fibril alignment in developing embryonic tendons in which fibrils are all aligned in the same axis (Birk and Trelstad, 1986, Canty et al., 2004, Kalson et al., 2015) but rather little is known about collagen deposition in a more complex ECM for example, as skin is developing or when dermal tissues are rebuilt after wounding, and this is largely due to technical difficulties in live imaging these processes in model organisms. Several methodologies have been used to visualise collagen within fixed tissues, including classic histological stains such as Masson's Trichrome (Mori et al., 2008), or immunohistochemical staining for collagen (Richardson et al., 2013). Transmission electron microscopy offers complementary opportunities for ultrastructural analysis of collagen deposition (Starborg et al., 2013). More recently, second harmonic generation (SHG) imaging using multiphoton microscopy (Ingman et al., 2006, LaComb et al., 2008, LeBert et al., 2016, LeBert et al., 2015) and indirect visualisation of collagen through fluorescently labelled collagen binding molecules (Boerboom et al., 2007, Megens et al., 2007) have begun to enable live imaging of collagen. For example, collagen dynamics have been followed in live tissues in vivo using SHG to study cancer cell/microenvironment interactions in mouse tumours (Sahai et al., 2005); the use of collagen mimetic peptides (CMPs) (reviewed by Li and Yu, 2013) has similarly enabled detection of collagen surrounding tumours within mice (Li et al., 2012). Although these approaches have offered indirect opportunities to image collagen in tissues, greater insights are expected to come from real time visualisation of collagen deposition and fibrillogenesis in a live, in vivo model organism expressing fluorescently tagged collagen. More recently, GFP*topaz* and mCherry-tagged collagen constructs have been generated for live imaging of collagen assembly in murine osteoblasts in vitro, in which the GFP-tag replaced the N-terminal propeptide of the collagen alpha2(I) chain (Lu et al., 2018). A transgenic mouse expressing GFP*topaz*-collagen under control of the 3.6 kb type I collagen promoter has also been developed (Kamel-ElSayed et al., 2015). We have chosen the zebrafish as a model system to probe collagen dynamics because of its genetic tractability and the optical clarity of the developing skin. By generating a transgenic zebrafish line expressing collagen I fused to GFP (or mCherry) we can begin to live image deposition of collagen during skin development, and also follow collagen deposition in repairing skin; and by crossing this transgenic fish with various existing transgenic fish lines we can, for example, probe the interplay between epidermal cells and collagen I deposition at the repair site.

## 2 Results and discussion

### 2.1 Zebrafish collagen I - GFP labelling

In order to live image collagen deposition in larval zebrafish skin, we generated an epidermal-specific GFP-collagen I transgenic zebrafish line. It was previously presumed that due to the complex fibrillogenesis process, tagging collagens with fluorescent proteins would be challenging because of the need to avoid perturbing quaternary structure and polymerisation capacity and thereby disruption of subsequent function. Basing our design on prior work of Lu and co-workers (Lu et al., 2018), we carefully considered the location for GFP insertion into the collagen molecule to ensure that the GFP label remains attached but does not significantly disturb collagen polymerisation and generation of functional fibrils within tissues (Kamel-ElSayed et al., 2015).

Zebrafish collagen I is composed of α1a, α1b and α2 protein monomer chains, typically forming α1a(I)α1b(I)α2(I) heterotrimers (Gistelinck et al., 2016, Morvan-Dubois et al., 2003). As for mammalian collagen I, the N-propeptide of zebrafish collagen a1(I) contains a von Willebrand factor type C (vWFC) like domain, whereas a2(I) lacks this domain. We did not want to perturb any potential function that the vWFC like domain might confer so we chose the α2(I) chain for GFP labelling.

**Fig. 1:**
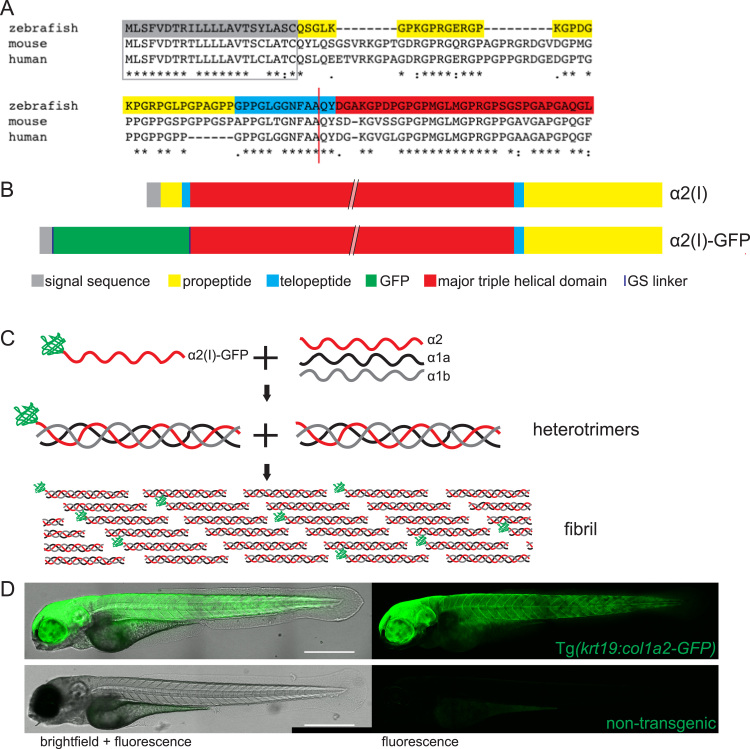
**Generation of a GFP labelled collagen I zebrafish line**. (A) The N-terminal regions of zebrafish, mouse and human collagen I α2 chains were aligned to determine the N-terminal proteinase cleavage site (red line) and identify the optimal GFP insertion site. (B) By inserting GFP in place of the N-terminal pro- and telo- peptide and removing the N-terminal proteinase site, GFP was retained on the α2 monomer. (C) GFP-tagged α2 trimerises with unlabelled α1a and α1b monomers and fibrillogenesis occurs with labelled and unlabelled trimers. (D) Tg*(krt19:col1a2‐GFP)* transgenic fish exhibit GFP labelling within flank skin when compared to control, non-transgenic zebrafish where only the gut shows faint autofluorescence. D is composed of a 4-image tilescan confocal image of a 4 dpf zebrafish. Scale bar = 0.5 mm.

The N-terminal region of human, mouse and zebrafish α2 peptide chains were aligned and compared to enable identification of the domain boundaries (Fig. 1A) (Dubois et al., 2002). The 22 amino acid signal sequences of all three species demonstrated high identity (mouse and zebrafish share 86%).

The N-terminal proteinase site is responsible for directing the cleavage of the pro- and telo- peptide from the major triple helical region which goes on to be utilised in trimer formation (Hojima et al., 1994). The sequence flanking the N-terminal proteinase cleavage site in all species was GNFAA|QY (with | denoting the scissile bond) (Fig. 1A). We removed this site by targeted mutagenesis to ensure retention of the GFP which we inserted upstream of this position.

Considering the primary structure of α2(I), we inserted GFP immediately downstream of the 22 amino acid signal sequence and upstream of the N-terminal proteinase cleavage site, removing the sequence between these two positions (Fig. 1B). Expressing the GFP-collagen I α2 as a transgenic insertion in addition to wild type unlabelled collagen I α2 enabled production of chimeric collagen I fibrils containing both unlabelled and labelled trimers thus restricting the number of GFP molecules present on the fibril (Fig. 1C), and reducing potential destabilisation of fibrillar structure (Lu et al., 2018).

### 2.2 Expressing the collagen I α2 – GFP fusion DNA constructs in zebrafish skin

The GFP gene was inserted into the chosen position within zebrafish *collagen I α2* cDNA, to generate *col1a2-GFP*. To enable correct folding of both collagen and GFP, a flexible glycine-serine linker was introduced at either end of the GFP. The functionality of the zebrafish collagen I–GFP fusion protein was first tested by driving expression, using a CMV promoter, within mouse fibroblasts in vitro. Fluorescent imaging of these cells did indeed reveal fibrillar collagen labelled with GFP (data not shown).

As described in the Methods, an expression construct was created by driving expression of *col1a2-GFP* using the *krtt1c19e* promoter (*krt19*) (Lee et al., 2014). *Krt19* drives expression in the basal epidermal cell layer which are the cells previously reported to be responsible for deposition of skin collagen I at early stages of zebrafish development (Fisher et al., 2003, Le Guellec et al., 2004, Li et al., 2011). Founder fish were generated, all of which appeared healthy and fecund, and which exhibited similar spatial expression, but variable GFP intensity between larvae. Casper zebrafish with reduced pigmentation (White et al., 2008) were used to aid in live imaging particularly since melanocytes migrate to wounds (Lévesque et al., 2013) and perturb both fluorescent and SHG imaging. Subsequent screening of F1 and F2 generation larvae for GFP expression at 4 dpf enabled selection of a transgenic fish line that demonstrated robust, bright and stable GFP-labelled collagen I fibrils within the larval skin (Fig. 1D). A similar cloning and selection strategy was followed to generate a complementary mCherry collagen I fish. We have previously quantified percentage of total GFP/mCherry labelling of α2(I) as less than 12% in similarly generated murine cells (Lu et al., 2018); in zebrafish studies this is more difficult to directly calculate due to lack of zebrafish-specific α2(I) antibodies but analysis of gene expression at 5 dpf using qPCR suggests GFP labelling in our brightest fish is approximately 36% (Fig. S1).

### 2.3 Collagen I within larval zebrafish skin develops into a regular orthogonal structure

**Fig. 2:**
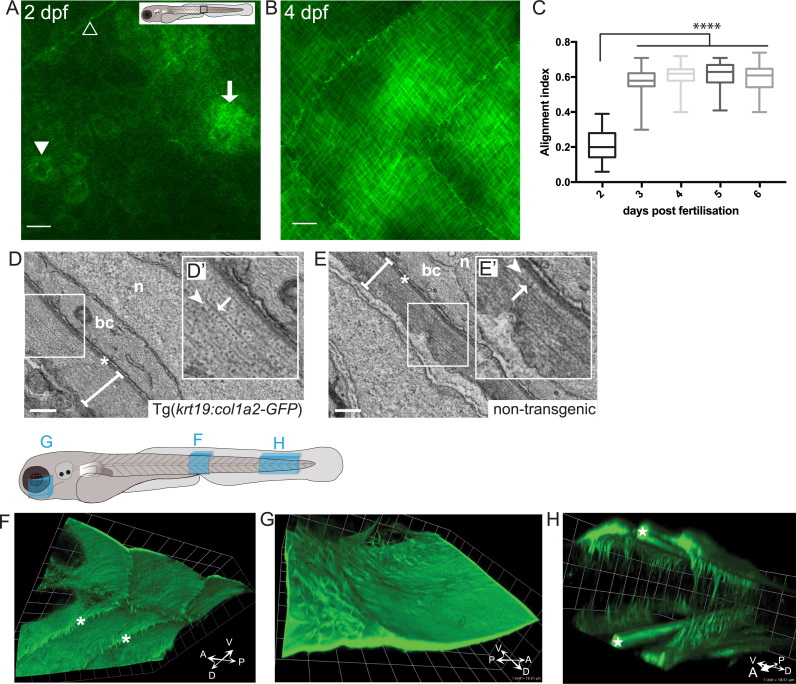
**Imaging the deposition of collagen beneath the embryonic epidermis**. (A) In the region of flank indicated by the box in larval schematic, the earliest GFP-collagen I is seen at 2 dpf in maximum projection confocal images of Tg*(krt19:col1a2-GFP)* transgenic zebrafish; GFP-collagen is seen within sporadic cells (arrowhead), and in some adjacent patches exhibiting the beginning of orthogonal patterning (arrow), as well as within myosepta (open arrowhead). (B) At 4 dpf orthogonal structure is fully evident. (C) Quantification of collagen alignment index (AI) over developmental time. Plotted as mean ± SD and analysed using a one-way ANOVA, ****p < 0.0001, n = 10–16 fish. (D, E) Transmission electron microscopy (TEM) of flank skin of 5 dpf Tg*(krt19:col1a2-GFP)* transgenic (D) and non-transgenic (E) larvae (with high magnification inset, D′, E′) reveals the orthogonal layering of collagen I; arrows indicate collagen fibril; arrowhead indicates adjacent orthogonal layer of collagen fibrils; n, nucleus; bc, basal cell cytoplasm; asterisk, basement membrane; line denotes collagen I layer. (F) GFP-collagen I tethers extend deep into the tissue of the 3 dpf fish as anchors to the underlying tissue within the myoseptum (asterisks); (G) Similarly, collagenous tethers link the epidermis to deep structures in the head, as for example around the eye; and (H) bilateral developing tail tendons are seen within the posterior-most portion of the developing tail (stars). Images in F, G and H are 3D reconstructions generated using Volocity, and correspond to regions indicated in the larval schematic above. Scale bars: A,B= 15 µm; C= 0.5 µm; F,G,H 1 unit= 18.51 µm; representative image of n = 3 fish imaged.

Weak expression of GFP-collagen first became apparent within the skin of Tg(*krt19:col1a2‐GFP*) larvae at 2 dpf and increased in intensity up to 5 dpf with particularly bright GFP expression observed first on the dorsal region of the head (Fig. 1D). At 2 dpf, GFP-collagen could be seen intracellularly in occasional epidermal cells and was also apparent as extracellular wispy fibrils adjacent to these cells (Fig. 2A). These early fibrils had no obvious orientation, just as observed by Le Guellec and co-workers when studying endogenous collagen deposition by Transmission electron microscopy (TEM) (Le Guellec et al., 2004). Previous gene expression studies indicate that endogenous *col1a2* mRNA is expressed from the 15-somite stage (Duran et al., 2015, Gistelinck et al., 2016), with protein expression following soon after (Le Guellec et al., 2004) and so GFP-labelled collagen I, driven from the krt19 promoter at 2 dpf, may be deposited where unlabelled wild type fibrils have already been laid down.

At 3 dpf we see GFP-collagen I fibrils beneath the flank epidermis beginning to remodel into a cross-hatched pattern with fibrils arranged perpendicular to each other, and by 4 dpf the fibril orthogonality was fully evident (Fig. 2B). Similarly, for mCherry-collagen I transgenic fish, a regular orthogonal pattern developed (Fig. S3B). Orthogonal GFP-collagen I labelling persists in the juvenile and adult zebrafish (Fig. S2); however, at these later stages labelling was restricted to the scale-layer. As expected, since the *krtt1c19e* promoter does not drive expression in the larval fin fold epithelium during zebrafish development (Lee et al., 2014), GFP labelling did not extend into the fins within larval transgenic fish. We believe that the GFP fluorescence reflects location of basal epidermal-derived collagen, however, some of the labelling within the Tg(*krt19:col1a2‐GFP*) fish might be ectopic due to the use of the strong non-collagen I, keratin promoter.

Utilising a modified version of the Fiji ‘Directionality’ plugin (Tinevez, 2010), a Fourier transform-based method, enabled analysis of the alignment of the fine collagen I-containing fibrils. Using the output from this plugin, further analyses (Sun et al., 2015) were performed which allowed us to compare alignment index (AI) between maximum projected images generated by confocal microscopy over a range of time points. This analysis demonstrated that AI increased from 2 dpf (0.21 ± 0.08) to 3 dpf (0.58 ± 0.08) reflecting increasing levels of collagen I orthogonality (Fig. 2C), which was then maintained from 4 dpf onwards.

TEM studies of our Tg(*krt19:col1a2‐GFP*) fish confirmed that the orthogonal fibrils within 5 dpf larval fish skin were orientated in plywood-like layers as previously demonstrated for wild type fish (Le Guellec et al., 2004) (Fig. 2D, E) suggesting that the GFP tag (and removal of N-terminal pro- and telo-peptide) does not significantly alter fibril patterning.

### 2.4 Myosepta, deep protrusions, and paired lateral muscle insertions are also labelled in GFP-collagen transgenic fish

As well as the orthogonal sub-epidermal collagen network, we also observed protrusions of collagen I extending from this meshwork deep into tendinous myosepta, separating the muscle blocks (Fig. 2F, S3A), as previously described in EM studies (Charvet et al., 2011). These data strongly indicate that the myosepta receive at least some contribution from epithelial cells although what proportion of the collagen in this structure might also be derived from fibroblasts (Bader et al., 2009, Kudo et al., 2004) cannot be determined from our studies.

In the tail of our transgenic fish we observe lateral tethers and bilateral, rod-shaped structures (Fig. 2H; also seen by second harmonic generation (SHG) imaging, Fig. S4) which we presume are developing tail tendons as previously reported in tuna fish (Shadwick et al., 2002). In addition, in the head there are numerous deep protrusions tethering the skin to underlying structures of the developing craniofacial skeleton (Fig. 2G) (Chen and Galloway, 2014).

### 2.5 GFP-collagen I labelling complements but is more informative than second harmonic imaging

We directly compared confocal images of the GFP-collagen I transgenic fish with images acquired from the same larvae by SHG (Fig. S5) and while both reveal some details of the orthogonal collagen structure, SHG imaging led to considerable interference from the deeper muscle tissue while GFP-collagen I labelling allowed creation of maximum projection images enabling us to observe the fibril structure at high resolution throughout the curved zebrafish skin.

We were concerned that addition of GFP at the N-terminal might subtly alter fibrillar structure, similar to that seen in Ehlers Danlos Syndrome Type VIIB (EDS VIIB), where the N-proteinase cleavage site is not present on collagen I α2 chains causing the retention of the N-terminal propeptide (Eyre et al., 1985, Holmes et al., 1993, Watson et al., 1992). However, our transgenic fish line does not appear to exhibit any obvious skin symptoms of EDS VIIB, such as skin laxity. That said, whilst the GFP-tagged collagen construct leads to appropriate in vivo localisation of the GFP-collagen fusion protein, and fibrils appear normal by TEM and SHG imaging approaches, we cannot exclude more subtle differences in trafficking, processing, secretion or crosslinking due to the GFP tag. However, even with these potential limitations, the construct clearly provides a powerful and useful tool for in vivo collagen imaging.

### 2.6 Following skin wounding, collagen I is initially deposited irregularly, but is subsequently remodelled back to an orthogonal pattern as in unwounded skin

**Fig. 3:**
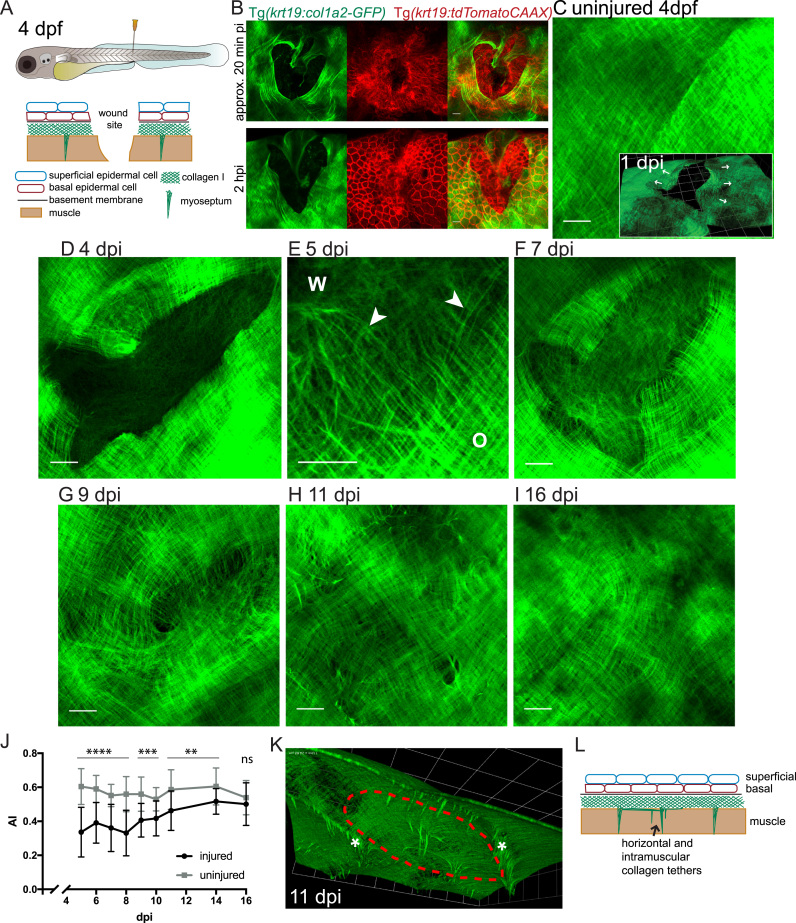
**Observing collagen deposition/remodelling following skin wounding**. (A) Schematic to illustrate the location/depth and tissues/layers involved in larval skin wounding. (B) Single and multi-channel images of wounds made to the flanks of Tg*(krt19:col1a2-GFP),* Tg*(krt19:tdTomatoCAAX)* double positive fish indicate how the epidermis (red cells) has partly healed in 20 min and completely healed over the denuded surface by 2 h post injury (hpi), whilst collagen I (green) remains absent in this region; see also Movie 1. (C-I) Max projection confocal images of Tg*(krt19:col1a2-GFP)* transgenic fish unwounded and wounded prior to imaging at the specified timepoints post injury (dpi); inset in C shows 3D reconstruction indicating collagen layer at 1 dpi with the collagen wound margin wrinkled where tension in this matrix meshwork has been released (arrows). E shows high magnification view of the interface between orthogonal (O) collagen, wound (W) margin and newly deposited wispy collagen fibrils (arrowheads) of a 5 dpi fish. (J) Quantification of collagen alignment index (AI) over the period of repair. For each timepoint mean of n = 7–12 wounds are plotted ± SD. ns, not significant, **p < 0.01, ***p < 0.001, ****p < 0.0001. (K) A 3D reconstructed, sub-epidermal view in the region of the repairing wound (red dotted line) indicates tethers extending both into muscle and between myosepta (asterisks). (L) Schematic to show tethers in addition to myosepta that remain at the healed wound site. For C-I Scale bars = 15 µm; inset in C and K, 1 unit= 24.68 µm.

To examine the dynamics of collagen deposition in a repairing skin wound we made stab wounds, with a hypodermic needle, to 4 dpf GFP-collagen larval fish (Fig. 3A) and observed GFP-collagen at various time points post-wounding. Immediately post-wounding confocal microscopy confirmed that sub-epidermal collagen I at the wound site was now completely absent (Fig. 3B). Its margins at the wound edge were raised and wrinkled as if released from tension at the time of wounding (Fig. 3C inset). Indeed, previous TEM studies of mouse embryo wound healing reveal a similar “elastic” retraction of the ECM following wounding (McCluskey et al., 1993). Crossing the GFP-collagen I fish against a line expressing membrane tethered tdTomato in the basal epidermal cells allows us to compare repair of the epidermis versus the epidermal-derived collagen at the wound site. The epidermal wound seals very rapidly, generally in less than an hour (Fig. 3B, S6, Movie 1), whereas the “defect” in the collagen I layer remained absent of collagen for up to 3 days post injury (dpi; Fig. 3D), with a clear edge delineating where the wound had been. Movies of several hours duration (Movie 1) can be made to capture collagen dynamics, without significant bleaching or ill health of the larva, but since collagen remodelling in the wound takes several days, the images we show are taken from several individual fish.

The following is the Supplementary material related to this article Movie 1.

Movie 1 **Migration of epidermal cells over collagen I layer post wounding**. Stab wounds were made to the flanks of 4 dpf Tg*(krt19:col1a2-GFP),* Tg*(krt19:tdTomatoCAAX)* double transgenic fish. Max projection confocal timelapse microscopy from approximately 0.5 to 17.5 h post injury indicates how wound re-epithelialisation is complete long before collagen matrix is repaired. Scale bar= 25 µm.

We observed new GFP-collagen I first being deposited in the wound gap from 4 dpi (Fig. 3D), after rapid closure of the epidermal wound (Fig. 3B, Movie 1). This new collagen was wispy and more randomly aligned than the orthogonal structure of adjacent unwounded skin collagen. Using the Fourier based methods described above, we quantified collagen orientation and showed that the AI (collagen alignment index) at 5 dpi was 0.34 ± 0.15 (compared to 0.60 ± 0.11 unwounded; Fig. 3J). Between 4 and 7 dpi, the quantity of irregular collagen in the wound domain increased, although, there remained a clear interface between this recently deposited wound collagen and the remaining collagen plywood structure surrounding the wound. At this interface we see wispy linkers, suggesting that new collagen may be deposited/fused onto the existing fibrils to extend the repairing matrix out into the wound domain (Fig. 3E).

By 9–11 dpi, the sub-epidermal collagen I within the wound site appeared to become more intimately bonded to the neighbouring unwounded matrix with some regions evolving towards a pre-wound orthogonal pattern (Fig. 3G,H), which was reflected in a gradual increase in AI throughout the timeframe of wound healing (Fig. 3J). By 16 dpi the wound site was increasingly difficult to locate because for many fish the collagen at the wound site had fully resolved back to near perfect pre-wound orthogonal pattern leaving no trace of a defect in the sub-epidermal meshwork, which was confirmed by comparable alignment indices (0.50 ± 0.13 injured, 0.54 ± 0.10 uninjured for 16 dpi) (Fig. 3I,J).

At sites where the myoseptum had been damaged, the “repair” collagen is not always perfectly regenerated in the typical chevron pattern as initially laid down during development, and these defects often remain beneath the healed skin. Separate from the myoseptum we also observe GFP-collagen deposits that lie horizontally just beneath the epidermis and appear to bind the adjacent undamaged myosepta together, and also deeper deposits within the muscle layer, suggesting that these might function as “adhesions” for the repairing epidermis at the wound site (Fig. 3K,L).

In mammalian skin repair, after wound-re-epithelialisation, the deeper dermal collagen that eventually forms a wound scar is formed of tight aligned bundles, rather than the more random, “basket-weave” fibrils of unwounded dermis, and this scar collagen is not subsequently remodelled to the original “unwounded” pattern (Cash et al., 2014, Eming et al., 2014). In adult zebrafish skin and heart, scar collagen is also deposited after damage, but is subsequently remodelled to leave behind a perfectly regenerated tissue without scar (Chablais et al., 2011, González-Rosa et al., 2011, Richardson et al., 2013). Further study of this regenerative healing of zebrafish tissues and a better understanding of how non-scar collagen is deposited beneath the epidermis may provide insights into how to modulate the mammalian repair process to reduce scarring.

We have demonstrated that by combining our GFP-collagen and mCherry-collagen fish with other established reporter fish we can exploit the unique live imaging opportunities available in larval fish to probe the processes involved in collagen deposition and remodelling. By crossing our GFP-collagen fish with one labelling basal epidermal cell membranes, we have been able to compare the healing of epidermis versus the re-establishment of collagen beneath the epidermis and reveal the dramatically different timecourse of healing for these two layers. Similarly, we have crossed the mCherry-collagen I fish against the ET37 (Parinov et al., 2004) enhancer trap line, labelling fibroblast-like cells (Lee et al., 2013) with eGFP, in order to observe the relationship between influx of these cells and epidermal collagen I deposition (Fig. S3C); as for re-epithelialisation, fibroblast-like cell influx occurs rapidly compared to matrix deposition. In the future, by crossing the GFP (or mCherry)-collagen-I lines against other fish lines, it will be possible to analyse the relationship between sub-epidermal collagen deposition and the wound inflammatory response (see Movie 2), or to compare the timecourse of deposition of the various matrix components of the basement membrane. Additionally, it would also be possible to study collagen dynamics within other tissues, by driving the expression of this GFP-collagen construct using other tissue-specific promoters.

Supplementary material related to this article can be found online at doi:10.1016/j.ydbio.2018.06.001.

The following is the Supplementary material related to this article Movie 2.

Movie 2 **Live imaging of interplay between wound macrophages and epidermal-derived wound collagen I**. 3D reconstruction of confocal timelapse imaging reveals macrophages migrating within the newly deposited epidermal-derived collagen I matrix in a Tg(*krt19:col1a2-GFP*), Tg(*mpeg1.1:mcherry*) double transgenic fish at 3 dpi. Even over a 15 h period very little new collagen is deposited indicating the slow timecourse of this process. Scale bar= 25 µm.

## 3 Materials and methods

### 3.1 Zebrafish lines and maintenance

Adult zebrafish (*Danio rerio*) were maintained and crossed as previously described (Westerfield, 2007). All experiments were conducted with local ethical approval from the University of Bristol and in accordance with UK Home Office regulations. Zebrafish lines utilised were, Tg(*krt19:TdTomatoCAAX*) (epidermis and collagen I) and Tg(*mpeg1.1:mcherry)* (Ellett et al., 2011) (macrophages and collagen I) which were out-crossed to Tg*(krt19:col1a2-GFP)* for dual-labelling experiments and ET37 (Parinov et al., 2004) which was out-crossed to Tg*(krt19:col1a2-mCherry*) to enable imaging of epidermal-derived collagen I alongside fibroblasts-like cells. All experiments were performed on Tg*(krt19:col1a2-GFP/mCherry)* homozygous fish and all fish were on a Casper background (White et al., 2008).

### 3.2 Generation of collagen I α2 – GFP DNA construct

The sequences of zebrafish collagen I α chains were compared to those of mouse and human using Clustalo alignments and literature searches (Dubois et al., 2002, Le Guellec et al., 2004) in order to establish conserved sequences and domain boundaries. Accession numbers for sequence comparison were BC071278.1, J03464 and X58251.1, for zebrafish, human and mouse respectively.

For generation of the GFP-collagen fusion protein, we followed the approach of Lu and co-workers in which the GFP tag replaced the N-terminal propeptide and telopeptide of murine alpha2(I) procollagen (Lu et al., 2018). Our strategy was to express the zebrafish GFP-collagen I α2 under control of a keratin promoter (Krtt1c19e (Lee et al., 2014)) to drive expression in basal epidermal cells. Plasmid containing zebrafish *collagen type I alpha 2* chain (*col1a2*) cDNA, pCMVSport6.1-*Zcol1a2*, was obtained from Source Bioscience. Mutagenic PCR, using primers Zcol1a007 (GGATCCacatgatgctaggtacgaagtcactg, *Bam*HI in capitals) and Zcol1a029 (cagtatgatggcgctaaaggacct), was used to remove the pro- and telo- peptide from the N-terminal of the *col1a2* gene and to insert a *Bam*HI restriction site at site of GFP insertion.

DNA amplified by PCR, using primers eGFP001 and eGFP003 (cgtgcgGGATCCatggtgagcaagggcgagg, actcgaGGATCCcttgtacagctcgtccatgcc, respectively, *Bam*HI in capitals) and template pME-GFP (Kwan et al., 2007), was purified by gel extraction from an agarose gel, digested and ligated into similarly *Bam*HI digested *col1a2*. Colony PCR was utilised to screen colonies for insertion of GFP in the correct orientation, generating plasmid pCMVSport6.1-*Zcol1a2-GFP*. Plasmid pME-*Zcol1a2GFP* was generated utilising a Gateway BP (Invitrogen) reaction along with pDONR221 and pCMVSport6.1-*Zcol1a2-GFP*.

A destination vector, pDEST-*krt19:col1a2-GFP*, was generated utilising pME-*Zcol1a2GFP,* p5E-*krtt1c19e*; (Lee et al., 2014), p3E-pA, pDESTTol2pA2 from the Tol2kit (Kwan et al., 2007), in a LR Clonase II Plus enzyme mediated LR Gateway recombination reaction (Invitrogen). Sequencing confirmed generation of all plasmids.

### 3.3 Generation of collagen I α2 – GFP/mCherry expressing zebrafish

A 1 nl volume of expression vector pDEST-*krt19:col1a2GFP* construct at 62.5 ng μl^−1^, together with 50 ng μl^−1^ purified Tol2 mRNA was microinjected into Casper zebrafish one cell stage embryos, as previously described (Hall et al., 2007). Injected larvae were screened for GFP expression at 3–7 days post injection by fluorescent microscopy and GFP positive larvae were grown to sexual maturity and screened for germline transmission. By screening successive generations a transgenic line was established which stably expressed bright GFP labelled collagen I within the skin (Fig. 1D). An identical process was adopted for generation of a collagen I α2 – mCherry expressing line utilising a pDEST-*krt19:col1a2mCherry* construct as outlined in Supplementary Methods.

### 3.4 Wounding and live imaging of zebrafish larvae

Four days post fertilisation larvae were wounded with a 30 G hypodermic needle on their flank directly above the cloaca. For imaging, fish were mounted on their sides in 1% low‐gelling temperature agarose (Sigma), in a glass‐bottomed dish, filled with Danieau's solution with 0.1 mg ml^−1^ tricaine anaesthetic. A Leica TCS SP8 AOBS confocal laser scanning microscope attached to a Leica DMi8 inverted epifluorescence microscope equipped with a 65 mW Ar laser along with ‘hybrid’ GaAsP detectors was utilised to image larval fish. Fish were imaged using a 63 × 1.3 NA glycerol objective.

Second harmonic generation (SHG) microscopy was performed using a Leica SP8 AOBS confocal laser tandem scanning microscope attached to a Leica DM6000 upright epifluorescence microscope with tunable Spectra Physics DeepSee dual beam pre-chirped 680–1300 nm multiphoton laser, set to 880 nm for SHG. A 25x HC Fluotar 0.95 NA water-dipping objective was used.

Imaging of wounded juvenile fish was performed on the same Leica SP8 AOBS confocal microscope but using lasers for single photon excitation (Argon and 561 nm) along with the internal ‘hybrid’ GaAsP detectors and transmitted light detector. A 25x HC Fluotar 0.95 NA water-dipping objective was used. Leica microscopes were interfaced with the LAS-AF software (Leica Microsystems, Wetzlar, Germany). Timelapse microscopy to generate movies of wound closure commenced 30 min or 3 days post injury with images taken every 10–30 min for 15–17.5 h. Images were processed using Fiji using maximum projection, 3D viewer for 3D reconstructions (Schmid et al., 2010) and bleach correction plugins (Miura et al., 2014).

### 3.5 Analysis of collagen fibril orientation

Maximum intensity and 3D projections were performed using Fiji (Schindelin et al., 2012) or Volocity (PerkinElmer). Analysis of collagen fibril orientation was performed using the ‘Directionality’ plugin (Tinevez, 2010) for ImageJ (Schneider et al., 2012) with amendments to split the results into angle ranges of 0–90 and 90–180 degrees. Also added to the plugin was an algorithm to report Alignment Index (AI) (Sun et al., 2015) for each of the angle ranges calculated using the following equation,

$$AI=\left|\frac{1}{N}\sum_{i=1}^{N}\left(2cos^{2}\left(\theta_{i}-\theta_{th}\right)-1\right)\right|$$

where $\theta_{i}$ is an angular measurement, $\theta_{\mathrm{th}}$ is the mean orientation angle and N is the total number of angular measurements. An AI= 1 would reflect no angular dispersion and thus full fibril alignment whereas AI= 0 would indicate an entirely random alignment of fibrils. Prior to calculation of AI, background was subtracted from the angular histogram to improve signal to noise. The plugin and source code are available within supplementary information.

Output from this plugin was further analysed using one-way ANOVA with Tukey's multiple comparisons test for developmental measurements and *t*-tests for wounded versus unwounded timecourse (Graphpad Prism 7.0).

### 3.6 Electron microscopy

Culled larval zebrafish were fixed in 0.05 M sodium cacodylate buffer (pH 7.2) containing 4% (v/v) glutaraldehyde, 1% (v/v) paraformaldehyde, 1 mM magnesium sulphate and 1% sucrose overnight at 4 °C. Ultrathin sections (70 nm thick) were prepared for electron microscopy as described previously (Canty et al., 2004).

## Acknowledgements

For our imaging, and image analysis support we thank The Wolfson Bioimaging Facility, Bristol University, and the MRC funding of a pre-clinical In-vivo functional imaging platform (multiphoton microscope) for translational regenerative medicine, as well as the Elizabeth Blackwell Institute, through its Wellcome Trust ISSF Award. We also thank all in the Martin Lab, as well as Rebecca Richardson for feedback as this MS was being drafted and Alex Greenhough for assistance in qPCR. This work was funded by Wellcome Trust Investigator awards to PM (097791/Z/11/Z) and KEK (110126/Z/15/Z), and NIH awards to SLD (R01AR051517 and P01AG039355).

## Declarations of interest

None.
