# Supplementary figures and images for: Live imaging of collagen deposition during skin development and repair in a collagen I – GFP fusion transgenic zebrafish line
Source: Dev Biol. 2018 Sep 1;441(1):4–11. doi: 10.1016/j.ydbio.2018.06.001 (PMC6080847; doi:10.1016/j.ydbio.2018.06.001)

## Supplementary figures

Fig S1

A

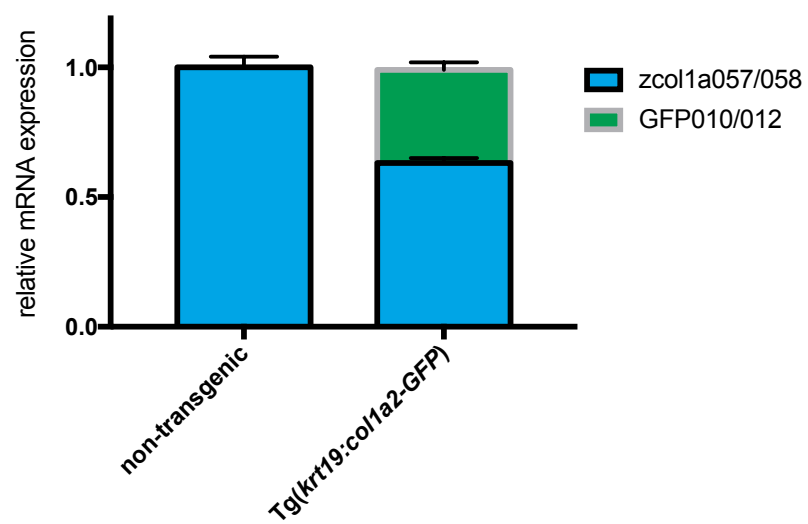

Fig S2  
A 28 dpf

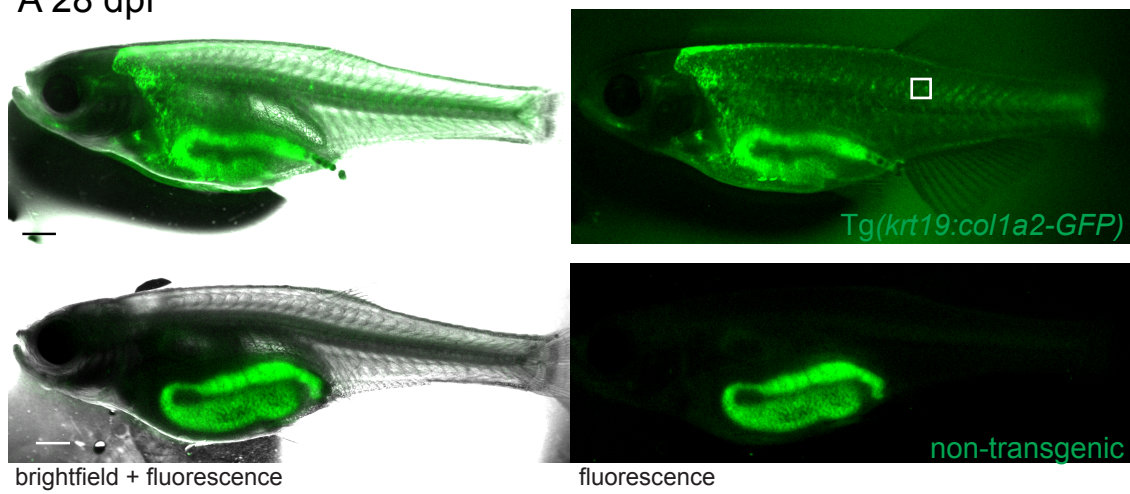

B

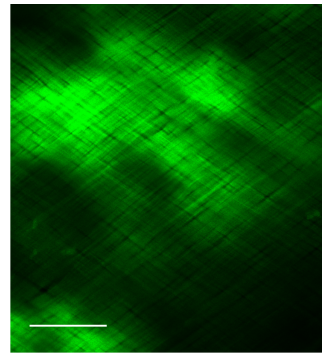

C 6 mopf

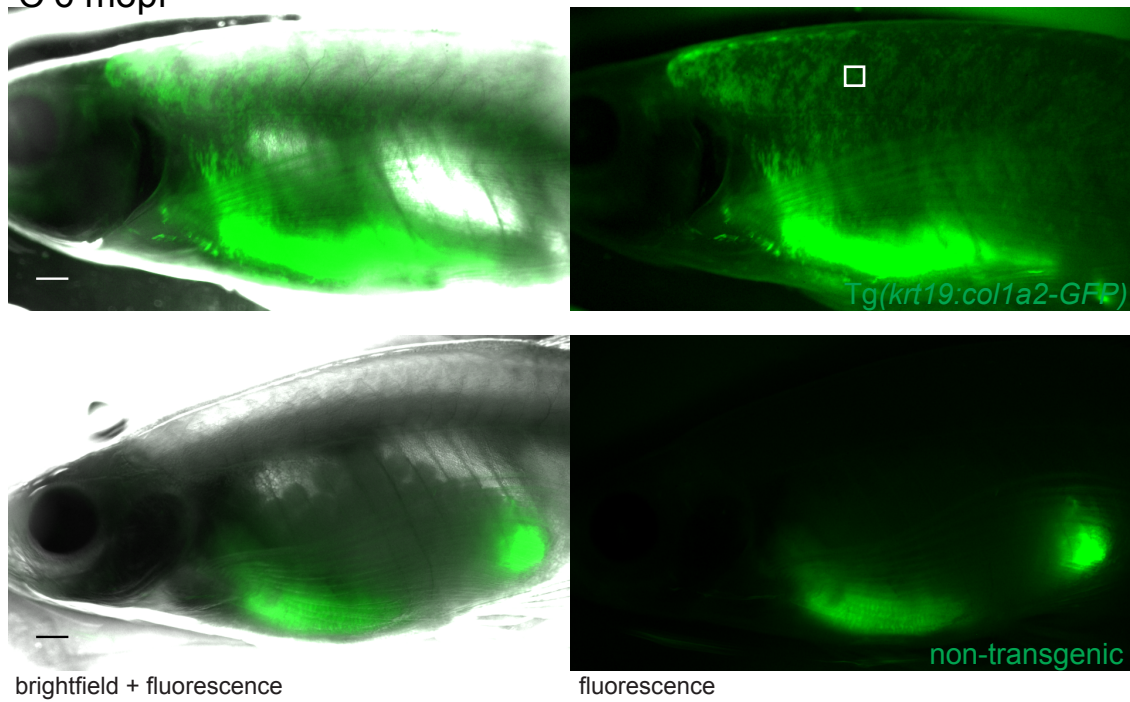

D

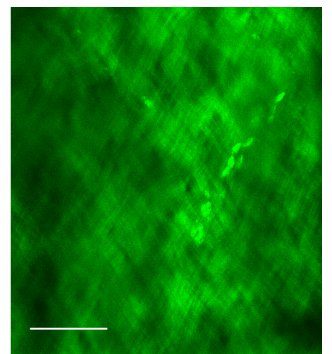

Fig S3

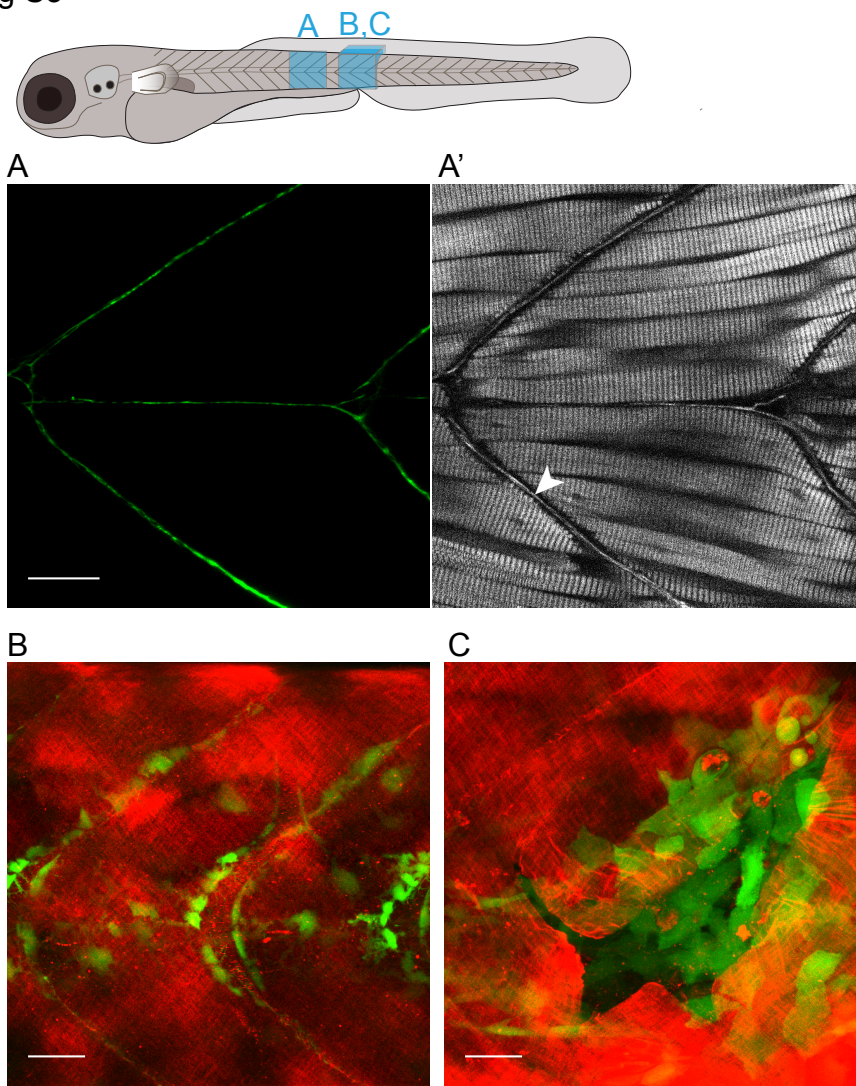

Fig S4

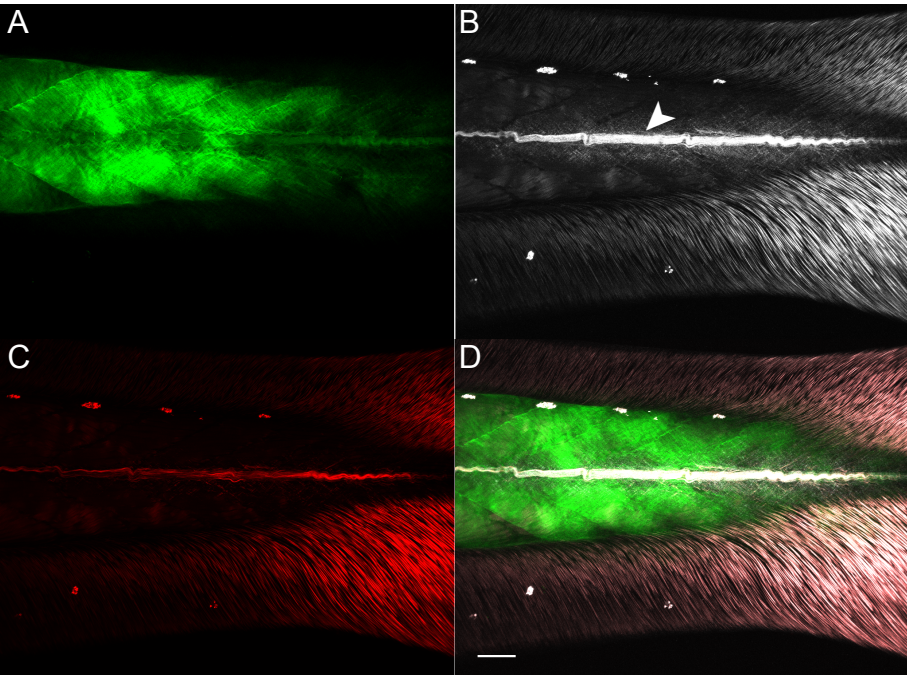

Fig S5

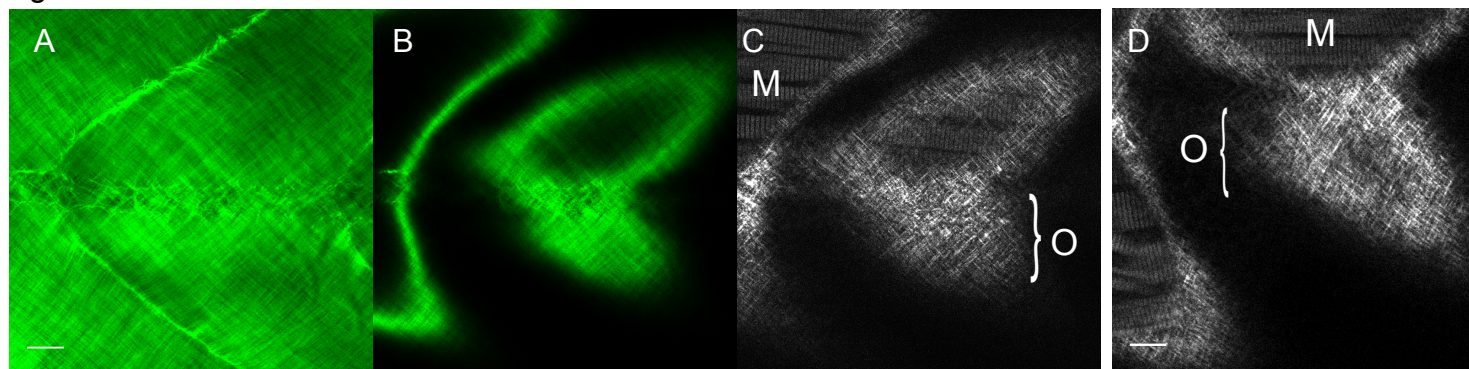

Fig S6

A 4 dpi

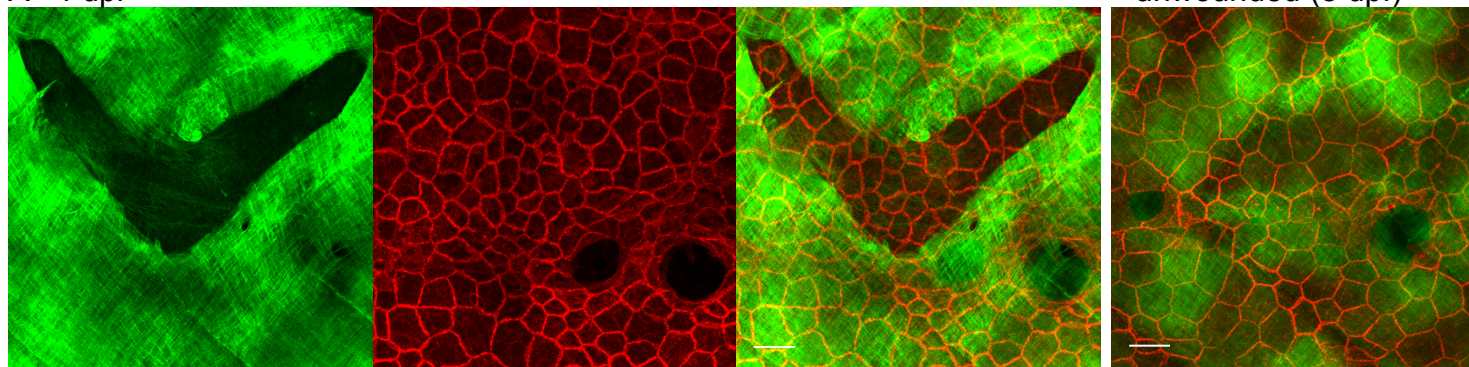

B 7 dpi

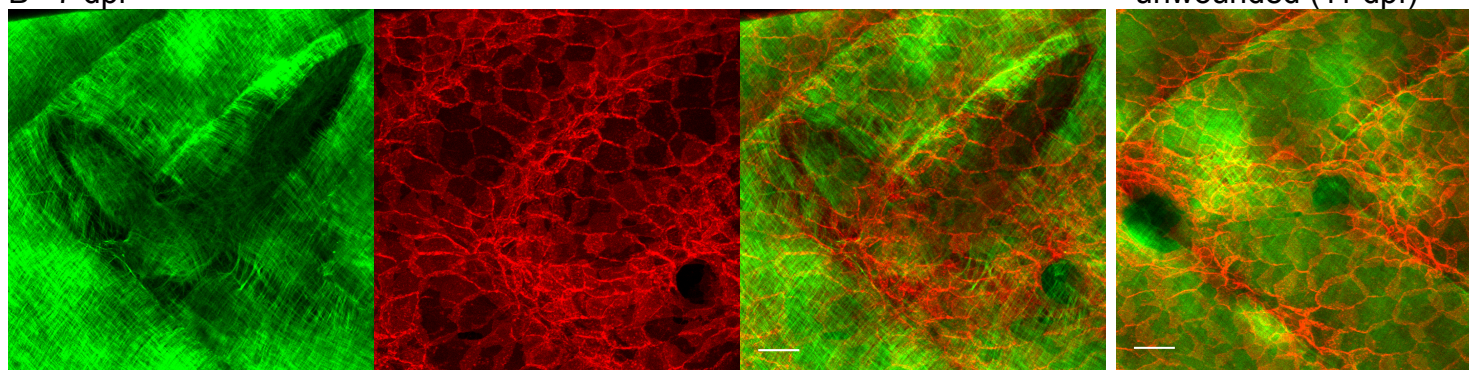

C 11 dpi

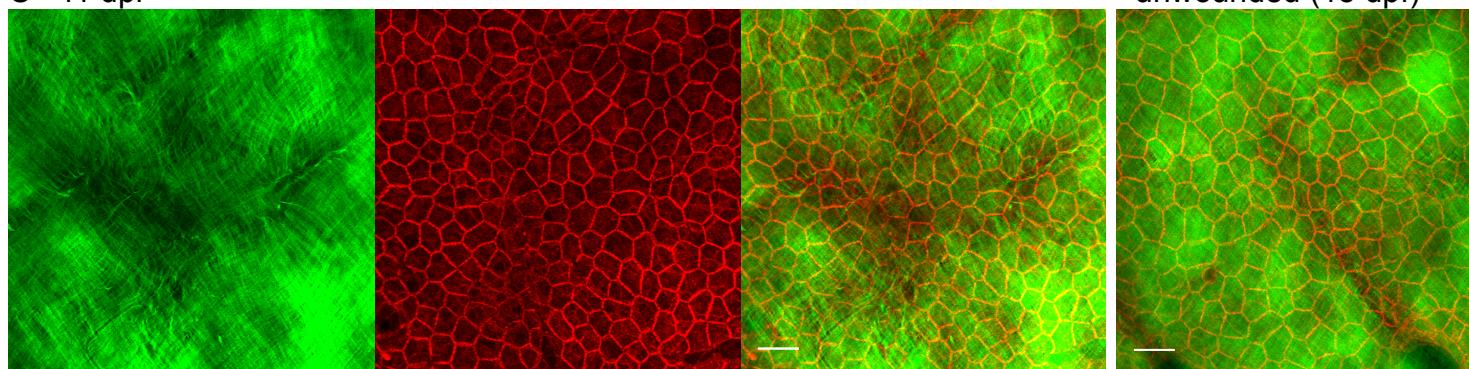

D 14 dpi

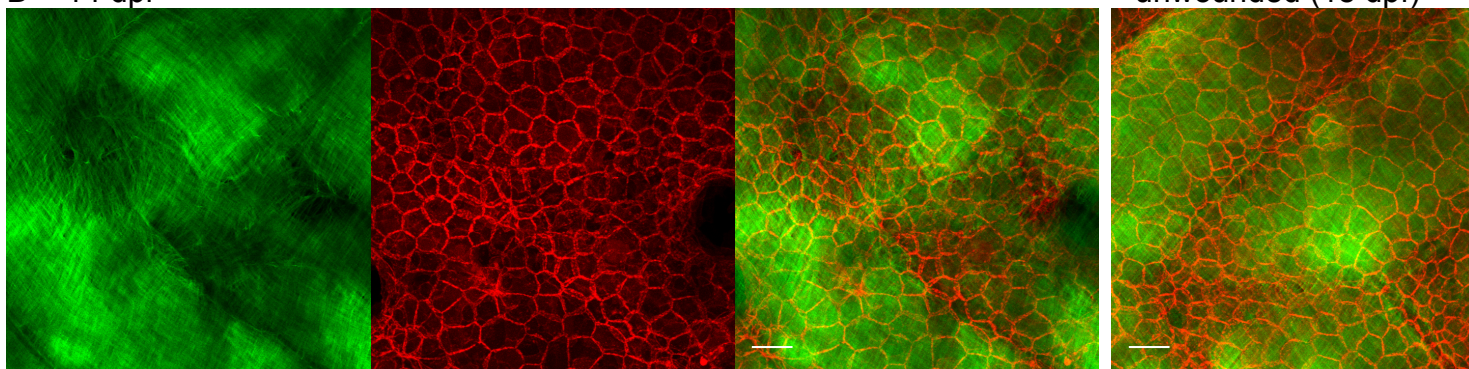

Supplement: Supplementary file 3 — Supplementary material [file mmc1.pdf]
