# Supplementary material for: Live imaging of collagen deposition during skin development and repair in a collagen I – GFP fusion transgenic zebrafish line
Source: Dev Biol. 2018 Sep 1;441(1):4–11. doi: 10.1016/j.ydbio.2018.06.001 (PMC6080847; doi:10.1016/j.ydbio.2018.06.001)
Supplement: Supplementary file 7 — Supplementary material [file mmc7.pdf]

Supplementary Information

Supplementary Methods

Generation of collagen I α2 – mCherry DNA construct

DNA amplified by PCR, using primers Zcol1a051 (cgtgcgACCGGTctggacacgcagtcaagggaacct, AgeI in capitals) and Zcol1a052 (actcgATCGATcggaacaatggacatgcttt, ClaI in capitals) and template pME-Zcol1a2-mCherry (generated as for pME-*zcol1a2GFP*), was purified by gel extraction from an agarose gel, digested with AgeI-HF/ClaI/DpnI and ligated into similarly AgeI-HF/ClaI/AnP digested and gel-purified pDEST-*krt19:col1a2-GFP*. Colony PCR was utilised to screen colonies for insertion of mCherry and production of pDEST-*krt19:col1a2-mCherry* was confirmed by DNA sequencing.

Generation of Tg(*krt19:tdTomatoCAAX*) transgenic zebrafish line

A stable Tg(*krt19:tdTomatoCAAX*) transgenic zebrafish line was produced using constructs generated using the Tol2 kit (Kwan et al., 2007), with the described *krttc19e* promoter fragment (Lee et al., 2014), along with pME-*tdTomatoCAAX* (a gift from Dr. Thomas Ramezani), followed by standard injection and screening procedures.

Imaging of juvenile and adult zebrafish

Fish were anaesthetised with 0.1 mg ml^{-1} tricaine in Danieau's solution, most of the liquid was removed and fish were laid out on a 10 cm dish. A MZ10F Leica widefield microscope equipped with a Leica DFC7000T camera and Leica LAS software was used to capture both brightfield and GFP fluorescent images. These images were overlaid using Fiji. Confocal imaging was performed as for larval zebrafish.

Analysis of gene expression

RNA was extracted from twenty 5 dpf zebrafish using TRIzol and isopropanol precipitation. DNase treatment and cDNA first strand synthesis was performed

using Maxima First Strand cDNA Synthesis Kit (ThermoFisher), using 0.4 μg RNA following the manufacturers protocol. Quantitative PCR was performed on cDNA using a SYBR Green PCR kit (Qiagen) in an Agilent MX3005P QPCR cycler using primer pairs zcol1a057/058 (AGGGACCAAAAGGACCCAGA and CAGCGAAGTTTCCACCAAGAC respectively) to amplify endogenous *col1a2* only and eGFP010/012 (CTCGTGACCACCCTGACCTA and GGCGGACTTGAAGAAGTCGT respectively) to amplify *eGFP* within *col1a2GFP* only. Amplification of *EF1a* (primers CTGGTTCAAGGGATGGAAGA and GAGACTCGTGGTGCATCTCA) was used as reference.

Supplementary Results

Figure S1. Analysis of *col1a2* and *col1a2-GFP* gene expression. Graph showing qPCR data indicating expression of *col1a2-GFP* is approximately 36% compared to *col1a2* in zebrafish larvae at 5 dpf.

Figure S2. GFP expression in juvenile and adult GFP-collagen transgenic zebrafish. (A) Widefield bright field and fluorescent images of 28 dpf and (C) 6 month pf (mopf) GFP-collagen I fish showing they remain GFP positive, whereas only the gut is autofluorescent in non-transgenic fish. Region imaged by confocal indicated by white box. (B, D) Maximum projection confocal images demonstrate the GFP-collagen I is still orthogonal in nature, but it is located in the scale-layer. Scale bars: A = 1mm; B,D = 25 μm.

Figure S3. Epidermal-derived myoseptal collagen I structures and relationship of epidermal-derived mCherry-collagen I to invading ET37 fibroblast-like cells (A) Equivalent single z-plane confocal and SHG (A') image of a 10 dpf Tg(*krt19:col1a2-GFP*) zebrafish, indicating myoseptal labelling (arrowhead in A'). (B,C) A maximum projection confocal images of Tg(*krt19:col1a2-mcherry*), ET37 double transgenic zebrafish to show relationship of epidermal-derived collagen I (red), with influx of fibroblasts-like cells (green) to wound, just prior to (B) and 2 dpi (C). Scale bars = 25 μm.

Figure S4. Multiphoton imaging of developing tail tendon in 10 dpf GFP-collagen transgenic fish. (A) Maximum projection confocal image of GFP-collagen I transgenic zebrafish. (B) Maximum projection of forward second harmonic generation (SHG) microscopy image. Tail tendon indicated by arrowhead. (C) Maximum projection of backward SHG microscopy image. Developing actinotrichia in the tail fin are also labelled in B and C (but not A). (D) Overlay of GFP, forward and backward SHG.

Figure S5. Comparing fluorescent images of GFP-collagen I transgenic fish versus Second Harmonic Generation (SHG) imaging. (A) A maximum projection confocal image and (B) single z-plane image of a 10 dpf GFP-collagen I transgenic fish directly compared to a single z-plane signal from a second harmonic generation (SHG) microscopy image of the same fish (C). (D) A single z-plane SHG microscopy image of a non-transgenic zebrafish. M, muscle fibres; O, orthogonal collagen fibrils. Scale bar = 15 μm.

Figure S6. Migration of epidermal cells versus epidermal collagen I deposition post wounding. Still images from repairing wounds to the flanks of 4 dpf Tg*(krt19:col1a2-GFP),* Tg*(krt19:tdTomatoCAAX)* double transgenic fish to reveal relative time course of repair of the cellular versus matrix layers. Scale bar = 25 μm.
